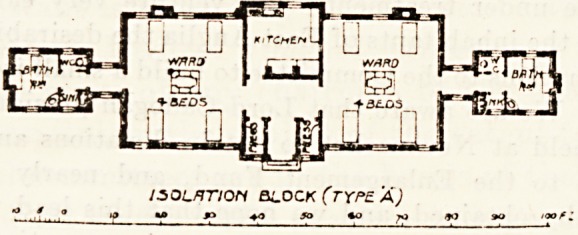# The City Hospital for Infectious Diseases, Liverpool

**Published:** 1906-11-17

**Authors:** 


### THE CITY HOSPITAL FOR INFECTIOUS DISEASES, LIVERPOOL.

Some years since, the City Council bought the Harbeck Estate at Fazakerley with the intention of building thereon a hospital for infectious diseases, and the project has now been carried out in a manner which seems to be eminently satisfactory. The cost of the estate was nearly £40,000, and it has an area of 118 acres. Being only five miles from the centre of the city, it is easily reached by cab, tram, or railway.

The buildings as finished consist of twenty-four separate blocks and cover about 35 acres of the site. The pavilions run north and south and thus obtain full benefit of the sunshine—when there is any. The pavilions are placed 100 feet apart, a space which is ample to obviate any risk of their over-shadowing each other.

The main entrance is in Longmoor Lane, and on the right of this is the medical superintendent's residence, a two-story block faced with Ruabon brick relieved with sills and cornices of white stone. The porter's lodge is on the left hand of the main entrance and opposite to the superintendent's residence, and the lodge is the administrative department, a three-

NEW CITY HOSPITAL FAZAKERLEY · LIVERPOOL ·

NURSES HOME ·

FIRST FLOOR PLAN ·

GROUND FLOOR PLAN ·

NEW CITY HOSPITAL FAZAKERLEY · LIVERPOOL ·

WARD PAVILION ( TYPE "A")

WARD PAVILION (TYPE "B")

story block. This contains all the usual offices, and a large number of bedrooms for the domestic staff. The Nurses' Home is similar in appearance to the administrative block, and is connected with it. It contains, on the ground floor, a spacious sitting-room, a dining-room, and a library, and also sleeping room for six night nurses. On the first floor are bedrooms for twenty-five nurses, and on the second floor for twenty-seven nurses. Each of these rooms is twelve feet long and nine feet wide and has an open fireplace. The principal rooms in this block face the south, and it should be added that all the nurses have separate bedrooms.

ISOLATION BLOCK (TYPE 'A')

Towards the east and west are the pavilions for the patients. There are nine ward pavilions and four isolation blocks: the total accommodation being for 350 patients.

The pavilions are of two types. Those of the first type are one-story buildings. Each is divided into two main wards, and these wards are 76 feet long, 26 feet wide, and 13 feet 6 inches high. Each ward contains twelve beds so that the superficial area per bed is 165 square feet, and the air space is 2,225 cubic feet. The wall space per bed is about 12 feet 9 inches. Between the wards are the vestibule, store-rooms, hall, and kitchen, and projecting beyond the latter are the separation single-bedded wards, so arranged, we are pleased to note, that cross-ventilation is provided for. In the large ward every bed has a window on both sides, and in addition to this there are ventilators under the beds and Sheringham valves over them. The warming is by ventilating stoves, and there are electric fans for distributing the warm air.

The sanitary annexes are placed at the extreme ends of the wards and they are properly arranged and properly cut off from the wards.

The other type of pavilion is very similar to the one described save that it has a verandah at one end, and therefore one of the sanitary annexes has to be placed on the side of the ward instead of the end.

The isolation blocks are also of similar design, but each block contains only eight beds divided between two wards, and there are no single-bedded separate wards, as such are scarcely needed in these blocks.

There is a well-fitted-up laundry provided also with a Lyons' disinfector. The discharging block is placed at the end of the estate near Lower Lane. The sewage is treated on the septic tank system.

The cost of the hospital buildings was £121,475, but this sum includes neither the site nor the "equipment." Without the equipment but including the site it seems to have cost about £460 a bed; truly a large sum.

The architect was Mr. Shelmerdine, of Liverpool, who was assisted in the planning by Dr. Hope, the medical officer of health. The contractors were Messrs. Morrison and Sons, of Wavertree.

## Figures and Tables

**Figure f1:**
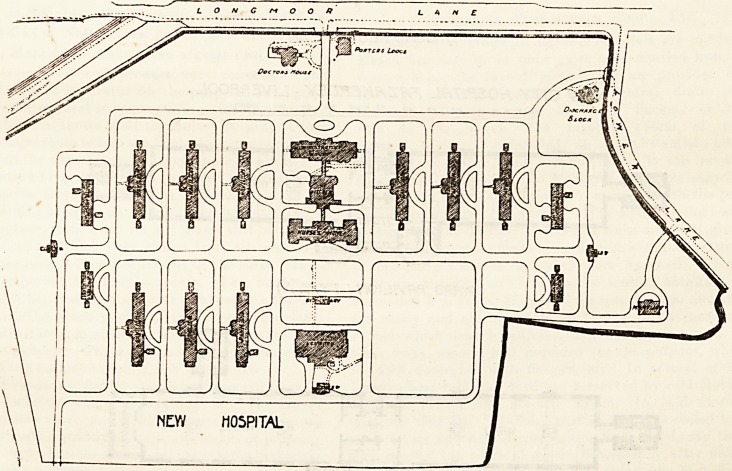


**Figure f2:**
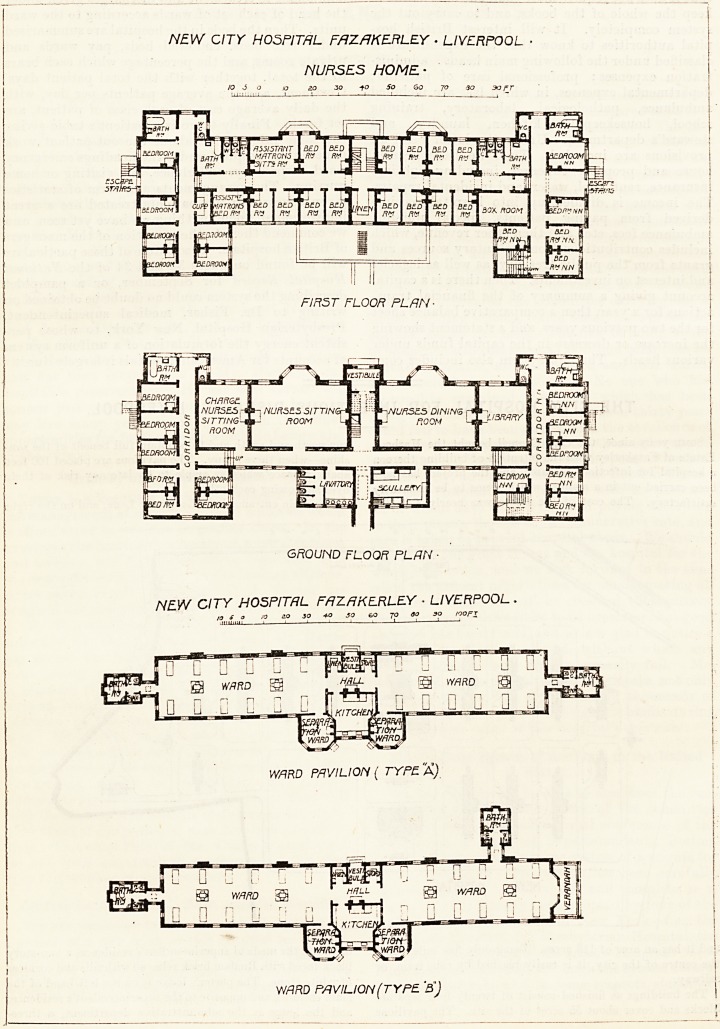


**Figure f3:**